# Potentiometric pH Nanosensor for Intracellular Measurements: Real-Time and Continuous Assessment of Local Gradients

**DOI:** 10.1021/acs.analchem.1c03874

**Published:** 2021-11-16

**Authors:** Mohaddeseh Aref, Elias Ranjbari, Juan José García-Guzmán, Keke Hu, Alicia Lork, Gaston A. Crespo, Andrew G. Ewing, Maria Cuartero

**Affiliations:** † Department of Chemistry, School of Engineering Science in Chemistry, Biochemistry and Health, Royal Institute of Technology, KTH, Stockholm SE-100 44, Sweden; ‡ Department of Chemistry and Molecular Biology, University of Gothenburg, Kemivägen 10, Gothenburg 41296, Sweden

## Abstract

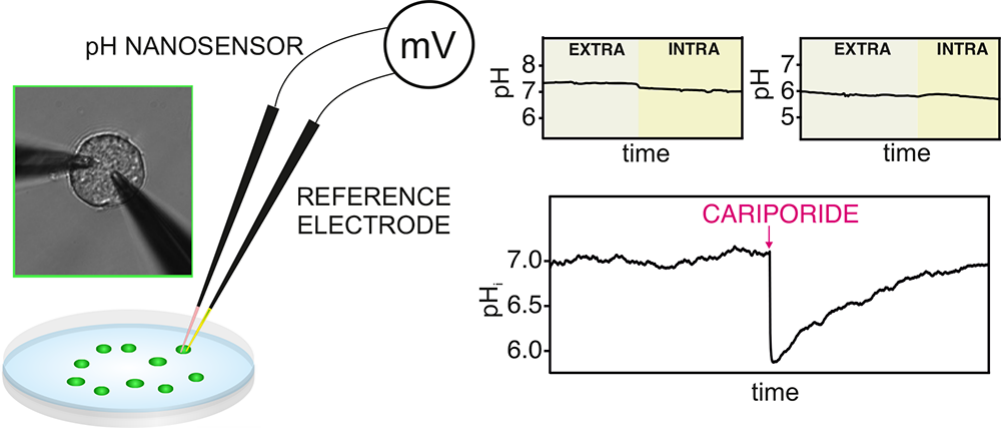

We present a pH nanosensor conceived for single intracellular measurements. The sensing architecture consisted of a two-electrode system evaluated in the potentiometric mode. We used solid-contact carbon nanopipette electrodes tailored to produce both the indicator (pH nanosensor) and reference electrodes. The indicator electrode was a membrane-based ion-selective electrode containing a receptor for hydrogen ions that provided a favorable selectivity for intracellular measurements. The analytical features of the pH nanosensor revealed a Nernstian response (slope of −59.5 mV/pH unit) with appropriate repeatability and reproducibility (variation coefficients of <2% for the calibration parameters), a fast response time (<5 s), adequate medium-term drift (0.7 mV h^–1^), and a linear range of response including physiological and abnormal cell pH levels (6.0–8.5). In addition, the position and configuration of the reference electrode were investigated in cell-based experiments to provide unbiased pH measurements, in which both the indicator and reference electrodes were located inside the same cell, each of them inside two neighboring cells, or the indicator electrode inside the cell and the reference electrode outside of (but nearby) the studied cell. Finally, the pH nanosensor was applied to two cases: (i) the tracing of the pH gradient from extra-to intracellular media over insertion into a single PC12 cell and (ii) the monitoring of variations in intracellular pH in response to exogenous administration of pharmaceuticals. It is anticipated that the developed pH nanosensor, which is a label-free analytical tool, has high potential to aid in the investigation of pathological states that manifest in cell pH misregulation, with no restriction in the type of targeted cells.

Intracellular pH (pH_i_) plays a vital role in modulating cell function, being an indicator of many diverse processes, such as vesicle trafficking, cellular metabolism, proliferation, and apoptosis, among others.^1^ Small alterations in the physiological balance of pH_i_ in response to exogenous signals (such as ischemia and nutrient deprivation events) likely indicate that hydrogen ions may function as second messengers to regulate cell signaling.^2^ As a result, the accurate and real-time monitoring of pH at the single-cell level has been recognized for the clinically useful information it reveals.^3−5^ Another important aspect is related to pH regulation in tumor cells, the monitoring of which is expected to provide new insights regarding the induction of tumor-specific apoptosis, while also aids to further advances in cancer chemotherapy.^6^

Today, the portfolio of analytical techniques available for the determination of pH_i_ primarily relies on spectroscopic measurements, including fluorescence imaging,^7−10^ and surface-enhanced Raman scattering.^11,12^ The main disadvantages of these approaches are that they often require extensive cell manipulation^13^ and, in particular for fluorescence studies, signal intensity is difficult to quantify with direct assays and is influenced by some experimental conditions and factors, such as dye localization, photobleaching and quenching.^3^ In contrast, electrochemical sensing is a label-free option for pH detection, and the electrode tip needed for measurements can be miniaturized down to nano-dimensions (nanotips). Additionally, this approach provides real-time and continuous signals with high spatial resolution.^3−5^ Another advantage is that the electrode tip can be tailored for the determination of different ions (*e.g.*, sodium, potassium, chloride, and metals) as well as biomolecules (such as glucose) inside and outside of a single cell.^14−17^

There are several options for the readout of electrochemical nanotips depending on the target. Potentiometry with a two-electrode system (indicator and reference electrodes) is traditionally used for non-redox active ions, whereas voltammetry/amperometry with a three-electrode system (indicator, reference, and counter electrodes) is widely dedicated to redox-active ions and biomolecules.^18,19^ In a simplified version of the voltammetry/amperometry mode, the reference and counter electrodes can be combined in a sole pseudo-counter/reference electrode.^20^ In particular for the determination of pH_i_, many publications reported ion-selective electrodes (ISEs) with potentiometric detection rather than voltammetry/amperometry. To the best of our knowledge, the very first attempts of pH_i_ measurements date back to the seventies and were based on glass microelectrodes containing an internal liquid contact, with relatively large tip dimensions and rather slow response time, which were limited by the configuration of the electrode (*e.g.*, tip geometry, the working principle of the glass membrane, and the use of inner-filling solutions as an internal reference).^21−23^ Seemingly, the use of an internal liquid contact has been the only approach used, until the time of this writing, in pH_i_ potentiometric measurements, hence limiting the reported applications to relatively big-sized cells, as following described.

In 1981, Simon and co-workers introduced the use of liquid membrane electrodes for pH_i_ measurements.^24^ The electrode was comprised of a glass micropipette (0.8–1.0 μm tip diameter) modified by injecting into the top of the shank (height ∼ 5 mm) an “ion-selective liquid” composed of the H^+^-selective ligand named tri-*n*-dodecylamine, sodium tetraphenylborate (cation exchanger), and *o*-nitrophenyloctylether (plasticizer). The authors demonstrated intracellular measurements in *xenopus laevis* oocytes (with diameter ∼ 1.3 mm) by inserting the pH electrode together with a reference microelectrode (filled with 3 M KCl solution) inside a single oocyte.^24^ Subsequent studies using similar electrodes revealed key information about the oocytes, including the mechanism and role of pH changes during meiotic maturation,^25^ the characterization of monocarboxylate transporter 1 and the renal electrogenic Na^+^/HCO_3_^–^ cotransporter as a result of changes in cytosolic pH or ion transport defect,^26−30^ the expression of the water channel aquaporin-1 to modulate CO_2_ permeability,^31^ and pH_i_ changes upon exposure to high (10–20 mM) and low (0.5 mM) levels of NH_3_/NH_4_^+^.^32^

Al-Hilli and Willander reported on a borosilicate glass capillary electrode (0.7 μm diameter) whose tip was modified with ZnO nanorods functionalized with proton and hydroxyl groups to provide a pH-dependent response.^33^ The pH was successfully measured inside a large single human fat cell (adipocyte with approximately 90 μm in size) *via* simultaneous insertion of the pH sensor and a Ag/AgCl reference microelectrode, providing a pH value of 6.81. Nanoelectrodes based on ZnO nanorods, nanoflakes, or nanowires were successfully used for intra- and extracellular measurements of other ions and biomolecules, including glucose.^17,34^ More recently, Pourmand and co-workers developed a nano-pH probe through physisorption of chitosan onto hydroxylated quartz nanopipettes (100 nm diameter) backfilled with 10 mM phosphate buffer saline solution at a pH of 7.0 as the inner filling solution.^3^ The protonation degree of the chitosan material was related to the pH of the medium in which it was located, which was reflected in a change of the current provided by the electrode interrogated with linear sweep voltammetry. The voltammetric nano-pH probe was used for pH_i_ measurements in non-cancerous and cancerous cell lines, including human fibroblasts (size of 10–15 μm), HeLa cells (40 μm), as well as breast cancer lines of MDA-MB-231 (∼20 μm) and MCF-7 (∼25 μm). The authors found that the pH of cancerous cells was slightly more acidic than fibroblasts (pH of 7.37 for fibroblasts, 6.75 for HeLa, 6.91 for MCF-7, and 6.85 for MDA-MB-231 cells).^3^

We describe herein electrodes developed using carbon-nanopipette ISE technology in an all-solid-state configuration to avoid the need for an inner-filling solution, in contrast to all pH sensors for pH_i_ reported at the time of this writing. We aim to circumvent the well-known disadvantages of the “liquid contact” *versus* “solid contact” potentiometric transduction, specially to avoid any restriction in the cell size that can be targeted with the electrode and toward more accurate real-time intracellular measurements. Regarding this latter, there is a serious risk of leaching effect from the inner solution (high ionic content) to the cell inside (low volume in small cells) that would entirely compromise the accuracy of the potentiometric measurements.^35^ The same reasoning applies to the reference electrode. On the other hand, the process of filling potentiometric micro- and nanoelectrodes with solutions is not straightforward, and the appearance of air bubbles usually prevents from an effective ion-to-electron transduction in addition to a lack of conductivity. Moreover, back-pressure issues may appear when the electrode is penetrating the cell membrane toward the inner part of the cell. In our approach, the nanoscale nature of both the pH indicator electrode and reference electrode makes it compatible with single-cell insertion. This provides an analytical tool with a high spatial and temporal resolution to cover the current demand in the field of pH_i_ measurements. We anticipate that the strategy proposed herein has the potential to develop new insights into many different body processes that manifest in pH_i_ changes, and therefore, a great plethora of cell-based applications are now open to be reached. In addition, the developed technology is compatible with the detection of other ions and applied to any type of cell.

## Experimental Section

### Development of the pH Nanosensor (Indicator Electrode)

**Figure 1:**
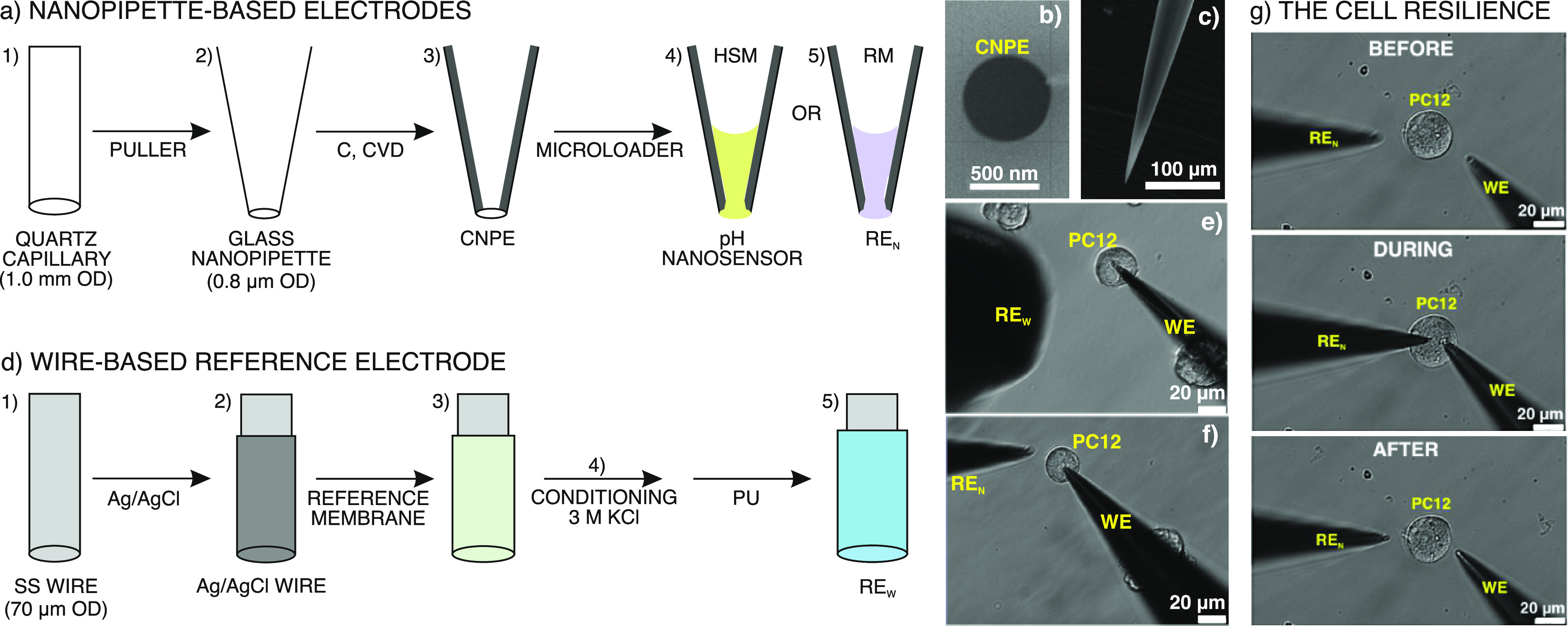
(a) Illustration of the procedure to prepare the pH nanosensor (WE) and the RE_N_. (b) Scanning electron microscopy (SEM) image showing the CNPE tip. (c) SEM image showing the length of the CNPE tip. (d) Illustration of the procedure to prepare the RE_W_. (e) Optical micrograph showing intracellular measurements with the WE and the RE_W_ positioned outside the PC12 cell. (f) Optical micrograph showing intracellular measurements with the WE and the RE_N_ positioned outside the PC12 cell. (g) Optical micrograph of a single PC12 cell before, during, and after insertion with the WE and RE_N_.

The pH nanosensor acted as the indicator electrode (WE) in the potentiometric measurements. The potentiometry readout was provided against a commercial Ag/AgCl reference electrode (RE_com_) in batch experiments for the characterization of the pH nanosensor response, and against two different homemade reference electrodes for cell-based measurements: RE_W_ (wire configuration) and RE_N_ (nanopipette configuration). In brief, the pH nanosensor was composed of a two-layer structure: a carbon film and the hydrogen-selective membrane (HSM, see Supporting Information).^36^ The carbon film has the dual purpose of providing large conductivity to the electrode substrate and as a solid contact to ensure a proper ion-to-electron transduction. Figure 1a illustrates the process for the preparation of the pH nanosensor. First, the nanopipettes (tip orifice O.D. 800 nm) were fabricated by pulling commercially available quartz capillaries (1.0 mm O.D., 0.7 mm I.D., Sutter Instrument, Novato, CA) with the CO_2_ laser pipette puller (P-2000/G, Sutter Instruments). Then, with the chemical vapor deposition (CVD) method, carbon is deposited readily on the inner wall of the nanopipette with an open channel in the middle because carbon sources (*e.g.*, methane) are effectively trapped in the tapered nanopipette, and result in carbon deposition due to their frequent collision with the inner wall.^37^ Moreover, a precise amount of carbon can be deposited by adjusting the duration of the CVD process to control the tip geometry to the nanopore dimension. A short deposition time (such as the 35 min used in our experiments) results in the coating of the inner wall of the nanopipette with a thin carbon film.^38,39^ Representative SEM images of the carbon nanopipette electrode (CNPE) tip are shown in Figure 1b,c. More experimental details are provided in the Supporting Information. Subsequently, the CNPE was backfilled with 10 μL of the HSM cocktail using Eppendorf Microloader pipette tips. Positive pressure was applied to the back of the electrode using Picospritzer II (General Valve, Fairfield, NJ) with 20 psi N_2_ for 10 s to ensure a good backfilling. The HSM cocktail contains a polymer, plasticizer, cation-exchanger, and hydrogen ionophore in THF so that, when the solvent is evaporated, the HSM membrane is formed in the nanopore of the CNPE. For this purpose, the membrane was left to dry for at least 4 h, and it was finally conditioned in 10^–3^ M HCl overnight. Electrical connections in the electrode were established by inserting a copper wire through the back end of the CNPE to make contact with the carbon layer.

### Development of the Reference Electrode (RE_W_ and RE_N_)

The procedures to develop the RE_W_ and RE_N_ are illustrated in Figure 1d,a, respectively. The first configuration (R_W_) consisted of an Ag/AgCl wire covered by a polyvinyl butyral (PVB) reference membrane (see Supporting Information).^40^ This sort of a membrane was demonstrated to provide a high and constant chloride concentration in all-solid-state reference electrodes, thus providing a constant potential that is independent of the background electrolyte.^40,41^ A stainless steel wire (70 μm diameter, Goodfellow, Cambridge, UK) was coated with the Ag/AgCl commercial paste. This layer was cured in the oven (120 °C, 10 min) and then, the reference membrane cocktail was drop casted (3 μL, three times) on top of the Ag/AgCl film. Each layer was allowed to dry for 20 min at room temperature before drop-casting the next one. Next, the last layer was dried for 4 h at room temperature before overnight conditioning in 3 M KCl. The electrode was dried at room temperature for 1 h and 4 μL of polyurethane solution (30 mg/mL) were drop casted on top of the modified RE_W_ and, finally, left it to dry in air for 4 h before re-conditioning in 3 M KCl. This outer membrane enhanced the potential stability of the RE_W_ and hinders any salt leaching.^42,43^

The second configuration (RE_N_) consisted of a CNPE modified with a PVB-based reference membrane cocktail, *via* backfilling of the CNPE with an Eppendorf Microloader pipette tip (same conditions as for the pH nanosensor). Notably, the PVB cocktail recipe was slightly different than that used for the RE_W_ to provide compatibility with the carbon filling of the CNPE.^41^ The membrane was left to dry for at least 1 h. Finally, the electrode was conditioned in 3 M NaCl for two weeks. The RE_W_ was used for extra- and intracellular measurements with an external position with respect to the cell under study (Figure 1e), whereas the RE_N_ was positioned differently (the WE and RE inside the same cell, two neighboring cells, and the WE inside and the RE outside the cell; this latter configuration is shown in Figure 1f) to investigate the configuration providing the most precise pH_i_ measurements. Importantly, the measured cells kept their normal morphology after insertion, as confirmed with a series of images of a single PC12 cell (Figure 1g) revealing excellent resilience before, during, and after electrode insertion. Both electrodes (WE and RE_N_) were functioning after several intracellular measurements, as confirmed with the maintenance of the calibration parameters before and after the cell insertion (data not shown).

## Results and Discussion

### Analytical Characterization of the pH Nanosensor

The potentiometric responses of pH nanosensors (electromotive force against the commercial Ag/AgCl reference electrode, EMF *vs* RE_com_) prepared with CNPEs of different tip dimensions (orifice diameters of 2.5 μm and 800 nm) were investigated in the pH range of 6.0 to 8.5, which includes the expected physiological range and pH levels related to other conditions, such as the pH expected in cancerous cells.^3^ The two different tip dimensions were selected to prove the versatility of the technology for intracellular measurements of cells of different sizes, as the tip must be fully inserted to provide a signal that relates only to the intracellular medium. The electrode with a diameter of 800 nm was indeed really miniaturized, aiming to reach very small cell sizes that have never been demonstrated at the time of this writing.

**Figure 2:**
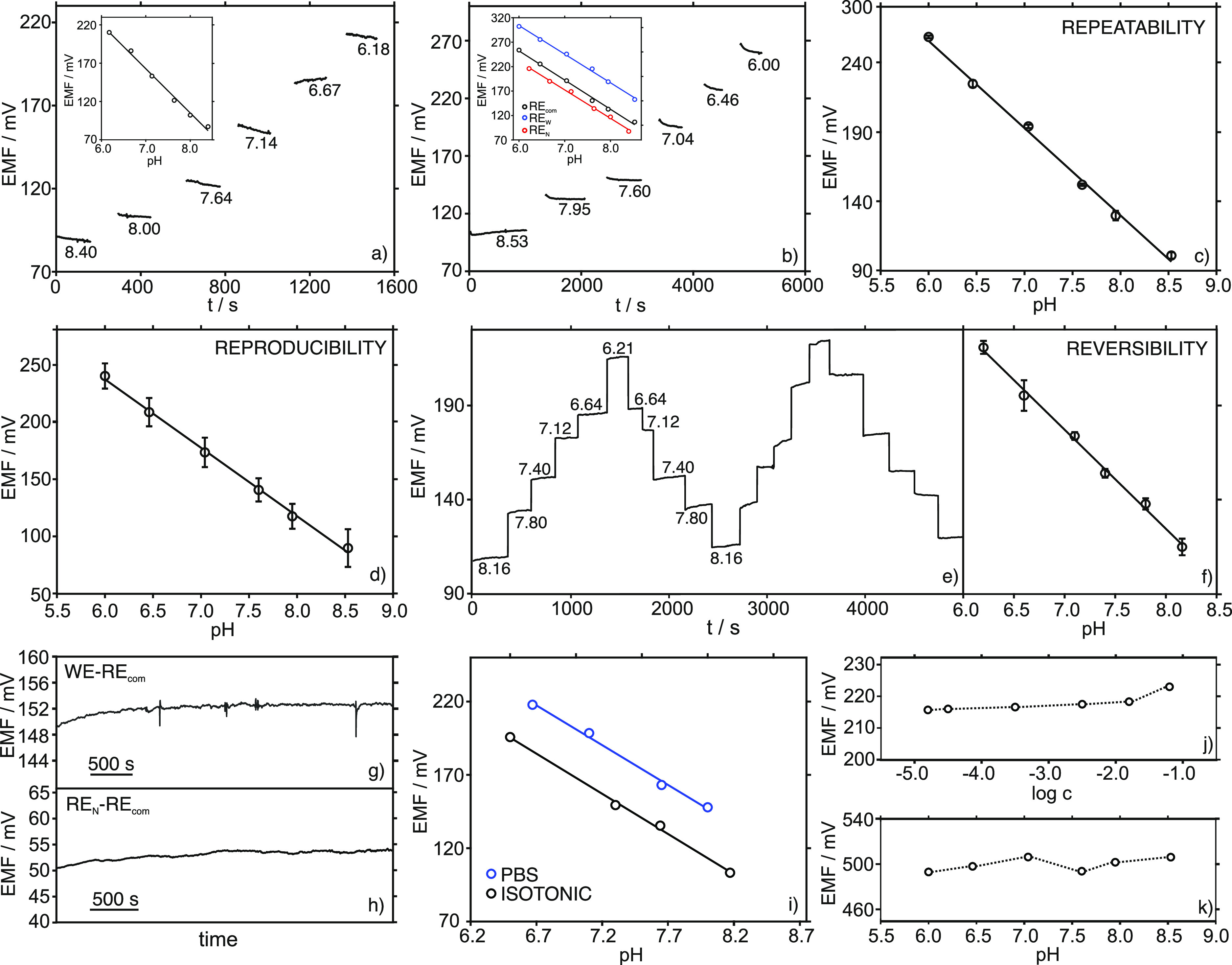
(a) Dynamic response of the pH nanosensor with O.D. = 2.5 μm against the RE_com_. Inset: corresponding calibration graph. (b) Dynamic response of the pH nanosensor with O.D. of 800 nm against the RE_com_. Inset: calibration graphs against the RE_com_, RE_W_, and RE_N_. (c) Average calibration graph observed for subsequent measurements (*n* = 3) with the same pH nanosensor (O.D. of 800 nm) against the RE_com_. (d) Average calibration graph obtained with three similar pH nanosensors (O.D. of 800 nm) against the RE_com_. (e) Reversibility study: dynamic potentiometric response corresponding to decreasing and increasing pH in the sample against the RE_com_. (f) Reversibility study: average calibration graph. (g) Medium-term response of the pH nanosensor (O.D. of 800 nm) in pH = 7.6 solution against the RE_com_. (h) Medium-term response of the RE_N_ in pH = 7.6 solution against the RE_com_. (i) Calibration graphs observed for the pH nanosensor against the RE_N_ in different media: phosphate buffer (PBS) and isotonic solution. (j) Average potentials measured with the RE_N_ against the RE_com_ at increasing KCl concentrations. (k) Average potentials measured with the RE_N_ against the RE_com_ at increasing pH.

Figure 2a,b presents an individual dynamic potentiometric trace for each pH nanosensor (*i.e.*, O.D. of 2.5 μm and 800 nm) at decreasing pH levels in the sample solution. When the calibration graph was plotted (potential *vs* pH), a Nernstian slope was obtained for both electrodes (−57.7 and −59.7 mV pH unit^–1^ for O.D. of 2.5 μm and 800 nm) against RE_com_. Further characterization was accomplished only with the smallest pH nanosensor (800 nm), and by assuming that similar behavior would be displayed by the larger electrode, as the preparation procedure was the same in both cases. Conveniently, the pH nanosensor with an O.D. of 800 nm is referred to as the pH nanosensor from now on.

Next, it was confirmed that the Nernstian response of the pH nanosensor was maintained independently of the reference electrode (*i.e.*, RE_com_, RE_W_, or RE_N_). Effectively, the calibration graphs obtained against the RE_W_ and the RE_N_ presented slopes of −58.1 and −55.5 mV pH unit^–1^, respectively. The change in the nature of the reference electrode was found to manifest in a shift of the potential for the intercept and thus, a displacement of the entire calibration graph (inset in Figure 2b) was observed.^41^

The potentiometric response of the pH nanosensor against the RE_com_ was investigated in terms of repeatability, reproducibility, reversibility, response time, drift, and interferences. The repeatability was evaluated from the results corresponding to three consecutive calibration graphs performed with the same sensor (Figure 2c), showing a coefficient of variation of 0.6% for the slope and 0.2% for the intercept. Between-electrode reproducibility was assessed by carrying out three calibrations using three similar pH nanosensors (Figure 2d), obtaining a coefficient of variation of 1.7% for the slope and 2.1% for the intercept. The variation observed for the intercept was slightly higher than that for the slope because the manufacturing method of the sensor involved a highly hands-on process, and therefore, a greater probability of differences between the prepared electrodes existed, which frequently resulted in different intercepts.^44^ However, this variation did not affect any further applications of the pH nanosensor for extra- and intracellular measurements provided that the electrode was calibrated before being used.

The reversibility of the pH nanosensor response was investigated by successive calibrations in which the pH was gradually decreased and increased in the sample solution. Figure 2e shows the dynamic potentiometric response observed for four consecutive calibrations and Figure 2f presents the average calibration graph, with a coefficient of variation of 1.4% for the slope and 9.1% for the intercept. These results indicate that it is convenient to recalibrate the electrode whether it faces relatively large pH variations (*i.e.*, close to two pH units) during a cell-based experiment. On the other hand, the absence of any significant medium-term drift (*ca.* 2 h) in the response of the pH nanosensor at a physiological pH of 7.6 (Figure 2g, 0.7 mV h^–1^), likely indicated that the variation of the calibration parameters observed in the reversibility study was primarily a consequence of drastic pH changes.

The medium-term drift (*ca.* 2 h) of the RE_N_ response versus the RE_com_ was also investigated at a physiological pH of 7.6, displaying an acceptable change of 1.8 mV h^–1^ (Figure 2h). Favorably, the RE_N_ can be used to calibrate the pH nanosensor in both buffered and isotonic solutions, showing Nernstian slopes in both cases (−56.1 and −56.3 mV pH unit^–1^, respectively; see Figure 2i). Moreover, the response of the RE_N_ for increasing KCl concentrations and pH was investigated (Figure 2j,k, respectively), presenting no significant influence. The final part of the analytical assessment of the pH nanosensor was based on a selectivity study including the major cations present in the intracellular medium: Na^+^, K^+^, Mg^2+^, and Ca^2+^. For these cations, the pH nanosensor displayed an almost negligible response, and thus, very low apparent selectivity coefficients were calculated using the separate solution method^45^
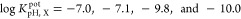
for X = Na^+^, K^+^, Mg^2+^, and Ca^2+^, respectively. These results confirmed the excellent selectivity already reported for membrane-based ion-selective electrodes based on hydrogen ionophore I (Sigma) for pH detection.^46^

### Investigation of the Positioning of the Reference Electrode in Cell-Based Experiments

For the sensing architecture to provide accurate potentiometric intracellular measurements, both the indicator (pH nanosensor) and the RE should be in principle placed inside the same cell. This is because of the intrinsic definition of the potentiometry technique, explained as follows. The EMF represents the difference between the potential occurring at the indicator–sample interface and that provided by the RE at zero current conditions.^47^ Ideally, the potential of each interface contained within the indicator-sample-RE system should be constant, except for that at the indicator–sample interface that must be designed for its potential to be dependent on the ion analyte concentration in the sample. Effectively, this is the role of the membrane (HSM) in the developed pH nanosensor, and thus, the related membrane potential (and indeed the EMF) is defined by the local equilibrium of ions present in the HSM and the sample.^48^

In the case of cell-based measurements, a pure indicator-sample-RE system is represented when the indicator and RE electrodes are introduced into the same cell. However, the situation changes when the RE is placed in the extracellular medium: an additional potential related to the cell wall (*i.e.*, cellular membrane potential) may influence the EMF measurements because of the formation of the indicator-sample-wall-RE system.^49^ Although not ideal, an alternative method is to position the indicator and RE in two neighboring cells. In this way, and assuming that the cellular membrane potential is identical in both cells, we reach a situation close to the simultaneous insertion of the indicator electrode and RE in the same cell. Thus, it is expected that the two cellular membrane potentials cancel each other because they will manifest as the same value but with a contrary sign in the potentiometry readout.^49,50^ Also, it is notable that no significant potential drop occurs between the indicator electrode and the RE by placing them as close together as possible.

**Figure 3:**
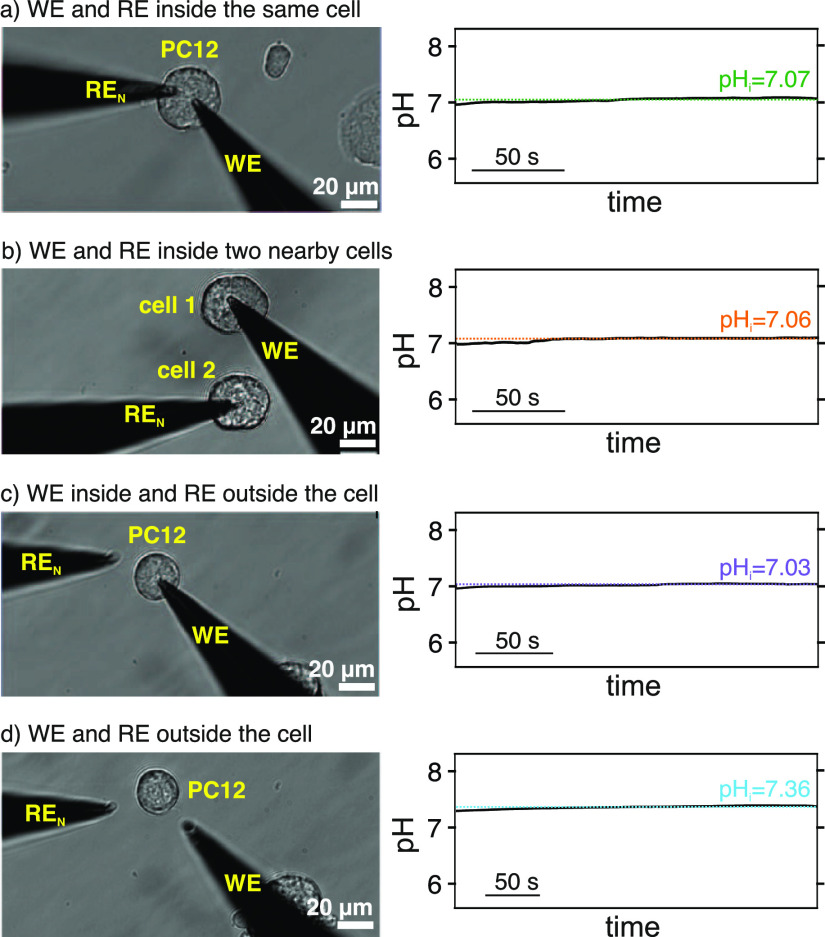
(a) Left: optical micrograph of the pH nanosensor (WE) and the RE_N_ measuring inside a single PC12 cell. Right: corresponding dynamic pH_i_ profile. (b) Left: optical micrograph of the WE and the RE_N_ measuring inside two neighboring cells. Right: corresponding dynamic pH_i_ profile. (c) Left: optical micrograph of the WE measuring inside a single PC12 cell with the RE_N_ outside and very close. Right: corresponding dynamic pH_i_ profile. (d) Left: optical micrograph of the WE and the RE_N_ while measuring extracellular pH. Right: corresponding dynamic pH profile.

Accordingly, we additionally investigated the profiles provided by the pH nanosensor when the RE_N_ is positioned outside the cell, inside the same cell, or a nearby cell. Figure 3 depicts images of the different positions together with the corresponding dynamic pH profiles observed once the pH nanosensor was inserted in the cell (Figure 3a–c) or outside the cell (Figure 3d). The average pH_i_ (during *ca.* 150 s) was found to be 7.07, 7.06, and 7.03 regardless of the position of the RE_N_ (Figure 3a–c). Advantageously, a similar pH value (7.03) was obtained when the RE_N_ was substituted by the RE_W_ operating outside the cell (pH = 7.05). These results revealed the adequacy of any of the three configurations to measure pH_i_, at least at the selected experimental conditions. Seemingly, the cellular membrane potential did not significantly influence the measurements, likely because the extra- and intracellular pH were similar. Nevertheless, this situation may not happen when measuring other ions that significantly contribute to the generation of the cellular membrane potential (in the order of −10 to −100 mV).^51^ For example, it is known that at physiological conditions, potassium is at a high concentration inside the cell (140–150 mM) and a low concentration outside the cell (3.5–5 mM).^16^ Also, some erroneous pH measurements may arise when manipulating the physiological balance of the cells and thus, pH changes are expected in both the extra- and intracellular media. All in all, the best way to ensure reliable potentiometric measurements is through assays including both the WE and RE inside the targeted cell, which is indeed feasible with the technological advances put forward in this paper.

The overall extracellular pH value in our experiments was confirmed *via* the immersion of the pH nanosensor and the RE_N_ in the extracellular medium. As shown in Figure 3d, an average pH of 7.36 was measured, which agrees with the pH in the original extracellular buffer solution (7.40, pH-meter). Moreover, the pH nanosensor and RE_N_ were used to determine the pH in the buffered culturing medium, which had a more complex background than the buffered extracellular medium. The analysis revealed a pH value of 7.45.

### Real-Time Monitoring of pH Gradients: from Extracellular to Intracellular Media

**Figure 4:**
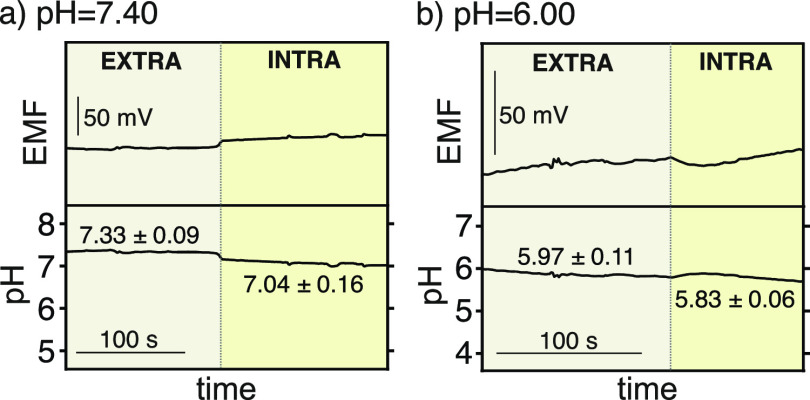
EMF traces (top) and the corresponding pH profiles (bottom) going from extracellular to intracellular measurements when the pH in the extracellular medium is fixed to 7.40 (a) and 6.00 (b).

The pH nanosensor and the RE_N_ were used for the real-time detection of the pH when spatially going from the extracellular to the intracellular media (with both the indicator and RE_N_ being inserted into the same single cell). Figure 4 presents the dynamic (and spatially-dependent) EMF and pH profiles when the extracellular medium was prepared at a pH of 7.40 (Figure 4a) and a pH of 6.00 (Figure 4b). As observed, the average extracellular pH in both experiments was close to the buffered solution: 7.33 ± 0.09 and 5.97 ± 0.11 for the extracellular media with a pH of 7.40 and 6.00, respectively. The pH_i_ was found to be slightly less than the extracellular pH in the first case (7.04 ± 0.16), but in the second case, the pH_i_ was very similar to the previous experiments with different positioning of the RE_N_. It was also very close to the extracellular pH (5.83 ± 0.06). These results show the capability of the developed potentiometric pH nanosensor to monitor how pHi is affected by changes in extracellular pH but also, to trace the pH from extra- to intracellular medium. Indeed, our observations are consistent with the investigations previously published by Dapreau and co-workers.^52^ Based on intracellular fluorescent pH indicators, it was found that an abrupt decrease of the pH in the external medium of synaptosomes (from 7.4 to 5.5) produced a decrease of the pH_i_ from 7.2 to 5.8 over 5 min.

### Continuous Monitoring of pH_i_ when the Cell is Exposed to the Cariporide Pharmaceutical

Several pathological states related to certain diseases, such as cancer, are known to be connected to pH misregulation in cells.^53^ It has been hypothesized that the cariporide drug serves as a highly selective target for anti-cancer therapy through the inhibition of NHE-1 (Na^+^/H^+^ exchanger isoform 1 that is abundantly expressed in PC12 cells)^54^ in tumor cells.^55^ In essence, the effect of cariporide can be associated with the activation of the apoptosis pathway. Through NHE-1 action, the inwardly directed Na^+^ gradient can drive a maximized extrusion of H^+^ and thus alkalinize the pH_i_ and acidify the extracellular pH.^56^ During myocardial ischemia, cariporide slows down the normalization of pH_i_ during reperfusion events triggered by ischemia-induced acidosis.^57^

The effect of cariporide administration to the extracellular medium on the pH_i_ of a single cell was investigated using the developed pH nanosensor (more details are provided in the Supporting Information). For this purpose, the pH_i_ in a PC12 cell was continuously monitored for a time frame of 1000 s before, during, and after the addition of a 10 μM concentration of cariporide in the extracellular medium. As a control experiment, the pH_i_ of a single PC12 cell was traced during the same time frame and without the addition of cariporide. The results were validated *via* the commercially available fluorometric assay based on the cell-permeable fluorescent indicator BCFL-AM.^58,59^ In all the cases using the pH nanosensor, six different cells were analyzed to obtain average pH values with their corresponding deviations (*n* = 6).

**Figure 5:**
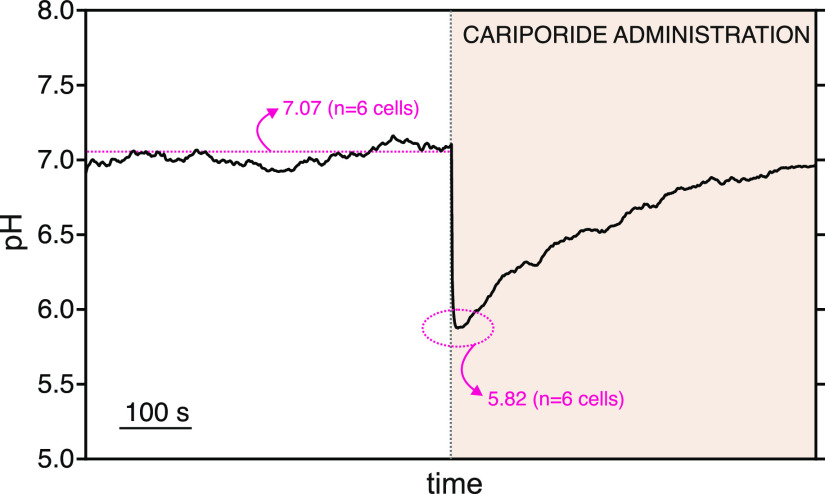
Dynamic pH_i_ measured with the pH nanosensor inside a single PC12 cell before, during, and after the addition of cariporide to the extracellular medium. Pink dotted lines: averaged pH_i_ (*n* = 6 cells) measured with the pH nanosensor.

Figure 5 depicts a representative pH_i_ profile provided by the pH nanosensor in the cariporide-based assays. While the pH_i_ remained constant in the control experiment (7.07 ± 0.01), it significantly (and rapidly) dropped from 7.05 to a pH of *ca.* 5.9 upon the introduction of cariporide into the extracellular medium. These values were averaged to 7.07 ± 0.05 and 5.82 ± 0.19, respectively, when the six cells were analyzed under the same conditions (the Mann–Whitney two-tailed test, *p* < 0.05). Then, the pH_i_ was found to gradually return to higher levels, reaching a value of *ca.* 6.9 after 8 min. This value was averaged to 7.00 ± 0.10 when the six cells were analyzed under the same conditions. The rapid pH_i_ decrease because of cariporide NHE-1 inhibition was indeed expected.This behavior agrees with the previous studies based on flow cytometry measurements using a pH-sensitive fluorescent sensor (BCECF-AM)^60^ Gao *et al.* found a similar decrease in pH_i_ induced by the addition of 10 μM cariporide to the extracellular medium.^61^ On the other hand, the explanation of the subsequent increase in the measured pH_i_ is not totally clear. Other authors previously observed this trend with other ion channel blockers and ascribed it to cell apoptosis,^3^ which resulted in the shrinkage of the cell body and exposing hence the sensor tip to the extracellular medium. However, we did not clearly identify the cell shrinkage in the microscope. Another hypothesis might be that the death of the cell makes the membrane immediately porous and hence, the extracellular fluid enters the cell altering the pH. Other mechanisms could be involved in the observed pH_i_ relaxation, the study of which is beyond the objectives of this work.

When fluorescence assays were used, unfortunately, it was not possible to detect the dramatic pH_i_ change experienced right after the cariporide addition. This was simply because of the handling methodology involved in the analysis, which requires more time than that observed for such a decrease in the pH_i_. Thus, the average pH_i_ measured after *ca.* 4 min of adding the drug was 6.67 ± 0.08, which indeed confirmed a decrease of the pH_i_ compared to the control experiment without any addition of cariporide (pH_i_ = 7.20). One of the advantages of using the pH nanosensor over the fluorescence assay is the provision of continuous pH_i_ tracing against punctual measurements. Rapid changes in pH_i_ can be discriminated with a higher temporal resolution. Furthermore, the developed methodology is a label-free option, and thus, there is no risk of affecting the medium, which may stress cells and alter the basal intracellular levels of the ions.^3,62^ The use of fluorescence dyes has not been recommended in cases where the course of pharmaceutical treatments needs to be studied because erroneous conclusions may arise as a consequence of side dye–pharmaceutical interactions that could change the cell physiology and activity.^3^

## Conclusions

The tailoring of a nano-sized potentiometric ion-selective electrode for single-cell pH_i_ measurements has been demonstrated in this work. The nanosensor is suitable for pH detection both at physiological levels and also at abnormal acidic conditions in cells. The fabrication approach has been developed *via* “solid contact” configuration for pH detection, but could be extended to any ion by changing the ionophore entrapped in the membrane in the nanopore of the sensor. In the particular case of pH measurements, our experiments demonstrate that the reference electrode necessary for the potentiometry readout can be placed inside the same cell, inside a neighboring cell, and also outside (and close to) the inspected cell. However, the need to insert both the indicator and reference electrodes into the same cell might be mandatory for other ions and certain pH circumstances. The pH nanosensor has been successfully applied to real-time monitoring of pH gradients from extracellular to intracellular media. Continuous tracing of pH_i_ inside a cell after it was exposed to the cariporide drug was also investigated. Preliminary results suggest that the pH nanosensor can provide temporal discrimination of a pH_i_ decrease and further be developed as a response to cariporide, in contrast to punctual measurements achieved with a commercially available fluorometry assays. This work opens new possibilities for obtaining clinically relevant data from single-cell potentiometric measurements of ion concentrations that are significant to cell activity. Moreover, the technological development put forward in this paper could be implemented in even smaller electrodes and thus, with no restriction in the targeted cell size.

## Supporting Information Available

Additional experimental details, materials, nanopipette preparation, potentiometric measurements, and drug-based experiments (PDF)
