# Supplementary material for: Potentiometric pH Nanosensor for Intracellular Measurements: Real-Time and Continuous Assessment of Local Gradients
Source: Anal Chem. 2021 Nov 16;93(47):15744–51. doi: 10.1021/acs.analchem.1c03874 (PMC8637545; doi:10.1021/acs.analchem.1c03874)
Supplement: Supplementary file 1 — ac1c03874_si_001.pdf [file ac1c03874_si_001.pdf]

Supporting Information for:

Potentiometric pH Nanosensor for Intracellular Measurements: Real-Time and Continuous Assessment of Local Gradients

Mohaddeseh Aref,† Elias Ranjbari,‡ Juan José García-Guzmán†, Keke Hu, ‡ Alicia Lork,‡ Gaston A. Crespo,† Andrew G. Ewing,‡ and Maria Cuartero*,†

† Department of Chemistry, School of Engineering Science in Chemistry, Biochemistry and Health, Royal Institute of Technology, KTH, SE-100 44 Stockholm, Sweden

‡ Department of Chemistry and Molecular Biology, University of Gothenburg, Kemivägen 10, 41296 Gothenburg, Sweden

*Corresponding author: Maria Cuartero (mariacb@kth.se)

KEYWORDS. pH nanosensor; Potentiometry; Single-cell measurements; pH gradients; Cellular activity

Table of contents

page

Experimental section S2
Reagents, solutions, materials and instruments S2
Nanopipette pulling S3
Chemical vapor deposition (CVD) S3
Potentiometric measurements S3
Intracellular pH measurements following drug administration conditions S3
References S5

EXPERIMENTAL SECTION

Reagents, solutions, materials, and instruments. All solutions were prepared using 18 MΩ·cm water from a Purelab Classic purification system (ELGA, Sweden). All chemicals were used as received. Hydrogen ionophore I (selectophore grade), sodium tetrakis[3,5-bis(trifluoromethyl)phenyl]borate (NaTFPB, > 98 % purity), bis(2-ethylhexyl)sebacate (DOS, ≥ 97 % purity), polyurethane (PU), tetrahydrofuran (THF, ≥ 99 % purity), polyvinyl butyral (PVB), silver nitrate, and cariporide were purchased from Sigma-Aldrich. Analytical grade chloride salts of calcium, magnesium, potassium, and sodium, as well as acetic acid/sodium acetate and sodium di-hydrogen phosphate/disodium hydrogen phosphate, were purchased from Sigma-Aldrich. Quartz capillaries (1.0 mm O.D., 0.7 mm I.D., 15 cm in length) were purchased from Sutter Instrument, Novato, CA. Ag/AgCl paste 50/50 (C2131007D3) was obtained from Sunchemical (UK). The isotonic saline solution used contained 150 mM NaCl, 5 mM KCl, 1.2 mM $MgCl_2$, 2 mM $CaCl_2$, 5 mM glucose, and 10 mM HEPES, with a final pH of 7.40 (close to physiological pH). A universal buffer based on different volume ratios of 0.1 M acetic acid/sodium acetate and 0.1 M sodium di-hydrogen phosphate/disodium hydrogen phosphate was used to provide standard solutions in the pH range of 6.0 to 8.5.

PC12 cells were provided by Lloyd Greene (Columbia University) and were maintained in RPMI-1640 medium (Lonza, Fisher Scientific, Sweden), supplemented with 10% donor equine serum and 5% fetal bovine serum in a 7% CO2, 100% humidity atmosphere at 37°C in an incubator. The cells were grown on collagen-coated cell culture flasks (collagen type IV, BD Biosciences, Bedford, MA) and were sub-cultured every 7 days. The medium was replaced every 2 days throughout the lifetime of the cultures. For single-cell experiments, PC12 cells were sub-seeded on collagen-coated culture dishes (collagen type IV, BD Biosciences, Bedford, MA) 4–5 days before the experiments.

The hydrogen-selective membrane (HSM) cocktail was prepared as a mixture of 1 wt.% hydrogen ionophore I (19 mmol kg^{-1} of membrane), 0.76 wt.% sodium tetrakis [3,5-bis(trifluoromethyl)phenyl]borate (NaTFPB, >98% purity, 8.6 kg^{-1} of membrane), 65 wt.% bis(2-ethylhexyl)sebacate (DOS, ≥ 97 % purity), and 33 wt.% polyurethane (PU) in 1 mL tetrahydrofuran (THF, ≥ 99% purity).[1]

The reference membrane cocktail for the RE_W was prepared by dissolving 78 mg PVB and 50 mg NaCl in 1 mL methanol.[2] The reference membrane cocktail for the RE_N was prepared by dissolving 78.1 mg PVB in 1 mL of methanol using the bath sonicator for 30 min. Then, 100 mg of NaCl and 50 mg $AgNO_3$ were added to the solution, forming a colloidal suspension of AgCl with a saturation of chloride anions from the excess in the NaCl.[3] Afterwards, the cocktail was exposed to a lightbulb (120 V/15W) for 10 min to facilitate the partial reduction of AgCl to metallic Ag, which is evidenced by a change of color in the colloid (from white to light purple).

Electromotive force (EMF) was measured with a high input impedance (1015) EMF 16 multichannel data acquisition device (Lawson Laboratories, INC). A Ag/AgCl reference electrode with a double-junction system was used for the characterization of the pH nanosensor (6.0726.100, Metrohm Nordic Sweden). Other instrumentation used included chemical vapor deposition (Carbolite Gero, UK), electrode holder (Axon Instruments, Union City, CA) and micromanipulators (Thorlabs Inc. Newton, NJ), inverted microscopes for live-cell imaging (IX81, Olympus, Japan), cell culture microscope (Leica Microsystems, Germany), fluorescence microplate reader (Fluostar OPTIMA, BMG Labtech, Durham, NC), laser pipette puller (P-2000, Sutter Instruments, Novato, CA), and scanning electron microscope (JEOL, JSM-7800F Prime, Field emission SEM, Japan).

Nanopipette pulling. Nanopipettes were pulled with a single line program and the parameters were as follows: heat 700, filament 4, velocity 30, delay 130, and pull 90. It should be noted that these values are instrument-specific, and parameters should be optimized for each instrument to obtain similar nanopipettes. Quartz material was selected because of its high melting point and chemically inert characteristics, which are necessary in the next electrode fabrication steps. Also, quartz does not interfere with any physical or chemical reactions regardless of the conditions.

Chemical vapor deposition (CVD). The pulled nanopipettes were placed in a quartz boat in the furnace under an argon flow and the temperature was ramped up to 980°C at a rate of 30°C min^{-1} (total experimental time of 35 min). At the elevated temperature, a methane/argon mixture (5:3) was passed through the CVD reaction chamber at a net flow rate of 480 sccm (standard cubic centimeters per minute) during heating. The furnace was then cooled under argon and the nanopipettes coated with carbon were recovered. Which such procedure, open carbon-nanopipette electrodes (CNPEs) were obtained by depositing a layer of carbon inside the pre-pulled quartz nanopipettes[63] using methane as the carbon source and argon as the protector.[64]

Potentiometric measurements. The potentiometric measurements were carried out at 37°C. The pH nanosensor and the corresponding RE (RE_{com}, RE_W, or RE_N) were connected to the potentiometer by a cable composed of electrical/crocodile clamps and BNC outputs. The calibration graphs with the pH nanosensor were performed in batch mode and against either of the three REs depending on the experiment. For example, the RE_{com} was used for the fundamental analytical characterization of the pH nanosensor and also to confirm that the homemade RE_W and RE_N provided a constant potential.

Before each intracellular experiment, the cell-cultured medium was removed from the culture dish and the cells were rinsed three times with a pre-warmed isotonic solution. Then, the cells were bathed in 4 mL isotonic solution and kept at 37°C on the microscope stage for the duration of the experiment. The potentiometric recordings from single PC12 cells were performed on an inverted microscope (IX81, Olympus, Japan) inside a Faraday cage. In particular for the measurements involving the pH nanosensor and the RE_N, bothvelectrodes were fixed above the inverted microscope by a holder (Axon Instruments, Union City, CA) and two different micromanipulators (Thorlabs Inc., Newton, NJ) were used for coarse and fine control of the electrodes' positioning. For each electrode, the tip was first placed close to the membrane of a single PC12 cell and then gently pressed to insert it into the cell.

Intracellular pH measurements following drug administration conditions. The pH nanosensor was inserted together with the RE_N inside the same PC12 cell and the potential (providing the pH_i) was recorded before, simultaneously to, and after the administration of cariporide to the extracellular medium (final concentration of 10 μM added from a cariporide stock solution in 0.1% DMSO, sterile PBS, pH of 7.4).

For validation of the pHi measurements in the presence of cariporide, PC12 cell cultures were exposed to a pH-sensitive fluorescent indicator (BCFL-AM) according to the protocol provided in the intracellular pH Assay Kit (MAK150, Sigma-Aldrich). To obtain the pH calibration curve, cell suspensions in the growth medium were washed and incubated with BCFL-AM dye within the pH range of 5.5–7.6. Then, the pH assay was run by monitoring the fluorescence at Ex/Em = 490/520 nm with the different pH solutions. To investigate the possible cariporide effect on the pH_i, the same concentration used in the potentiometric experiment (10 μM) was selected. One group of cells served as a negative control (without BCFL-AM) to evaluate the presence of intracellular autofluorescence. In the absence of the BCFL-AM dye, a negligible fluorescence value was observed for PC12 cells, which

was subtracted from the fluorescence intensity values obtained for pH calibration and cariporide treated cells.

REFRENCES

1. Canovas, R.; Padrell Sánchez, S.; Parrilla, M.; Cuartero, M.; Crespo, G. A., Cytotoxicity study of ionophore-based membranes: Toward on-body and in vivo ion sensing. *ACS Sensors* **2019**, 4, 2524-2535.
2. Kim, B.; Murray, T.; Bau, H., The fabrication of integrated carbon pipes with sub-micron diameters. *Nanotechnology* **2005**, 16, 1317.
3. Hu, K.; Jia, R.; Hatamie, A.; Le Vo, K. L.; Mirkin, M. V.; Ewing, A. G., Correlating molecule count and release kinetics with vesicular size using open carbon nanopipettes. *J. Am. Chem. Soc.* **2020**, 142, 16910-16914.
